# Blood-brain barrier transport of amyloid beta peptides in efflux pump knock-out animals evaluated by *in vivo* optical imaging

**DOI:** 10.1186/2045-8118-10-13

**Published:** 2013-02-25

**Authors:** Wandong Zhang, Huaqi Xiong, Debbie Callaghan, Hong Liu, Aimee Jones, Ke Pei, Dorothy Fatehi, Eric Brunette, Danica Stanimirovic

**Affiliations:** 1 Human Health & Therapeutics Portfolio, National Research Council of Canada, Ottawa, Canada; 2 Faculty of Medicine, University of Ottawa, Ottawa, Canada; 3 Current address: ApoPharma Inc., 100 Sussex Drive, Ottawa, Ontario, K1A 0R6, Canada; 4 Current address: The Hospital affiliated with the Shandong University of Traditional Chinese Medicine, Jinan, Shandong Province, China

**Keywords:** Alzheimer’s disease, Aβ peptides, Blood-brain barrier, Mdr-1a/b P-glycoprotein, Abcb1a/b, Abcg2, Optical imaging

## Abstract

**Background:**

Aβ transport (flux) across the blood-brain barrier (BBB) is thought to contribute to the pathogenesis of Alzheimer’s disease as well as to elimination of toxic amyloid from the brain by immunotherapy. Several BBB transporters have been implicated in Aβ exchange between brain parenchyma and the circulation, including efflux transporters P-glycoprotein/ABCB1 and BCRP/ABCG2. Here we describe an application of *in vivo* optical imaging methods to study Aβ transport across the BBB in wild-type or animals deficient in specific efflux transporters.

**Methods/Design:**

Synthetic human Aβ_1-40_ or scrambled Aβ_40-1_ peptides were labeled with the near-infrared fluorescent tracer, Cy5.5. The free tracer or Cy5.5-labeled peptides were injected intravenously into Abcb1^-KO^ or Abcg2^-KO^ mice or their corresponding wild-type controls. The animals were imaged prospectively at different time points over a period of 8 hours using eXplore Optix small animal imager. At the end of the observation, animals were sacrificed by perfusion, their brains were imaged *ex*-*vivo* and sectioned for immunofluorescence analyses.

**Discussion:**

After appropriate circulation time, the fluorescence concentration in the head ROI measured *in vivo* was close to background values in both wild-type and Abcb1^-KO^ or Abcg2^-KO^ mice injected with either free dye or scrambled Aβ_40-1_-Cy5.5. In animals injected with Aβ_1-40_-Cy5.5, the deficiency in either Abcb1 or Abcg2 resulted in significant increases in fluorescence concentration in the head ROIs 2 hours after injection compared to wild-type animals. Fluorescence decay (elimination rate) over 2–8 hours after injection was similar between wild-type (t_1/2_ = 1.97 h) and Abcg2^-KO^ (t_1/2_ = 2.34 h) and was slightly faster (t_1/2_ = 1.38 h) in Abcb1^-KO^ mice. *In vivo* time-domain imaging method allows prospective, dynamic analyses of brain uptake/elimination of fluorescently-labeled compounds, including Aβ. Deficiency of either of the two major efflux pumps, Abcb1 and Abcg2, implicated in Aβ trafficking across the BBB, resulted in increased accumulation of peripherally-injected Aβ_1-40_ in the brain.

## Background

Alzheimer’s disease (AD) is a chronic neurodegenerative disease characterized, among other neuropathological features, by the accumulation, aggregation and deposition of beta-amyloid peptides (Aβ peptides) in the brain [1,2]. Aβ peptides form oligomers, aggregates and plaques which are thought to contribute to synaptic dysfunction, neuroinflammation and neurodegenerative pathology in Alzheimer’s disease [1-4].

Mechanistic studies have generated a substantial body of evidence that brain accumulation of Aβ peptides is not solely due to their increased production in the brain, but also to reduced brain clearance and/or increased uptake from peripheral circulation [5,6]. Both latter processes are controlled by the polarized blood-brain barrier (BBB) receptors and transporters [7-10]. Blood-borne Aβ is taken up into the brain by the luminally-expressed endothelial receptor for advanced glycation end-products (RAGE) [11,12], whereas its brain efflux/clearance is largely mediated by the abluminal low-density lipoprotein receptor-related protein 1 (LRP1) [5,6,13,14]. A soluble form of LRP1 (sLRP1) is the major endogenous peripheral Aβ 'sink' that sequesters some 70 to 90% of plasma Aβ peptides [5]. Recent evidence also implicated key ABC family BBB transporters in Aβ trafficking between brain and circulatory compartments; luminal efflux transporter ABCG2 has been shown to prevent blood-borne Aβ from entry into the brain [8,15,16], whereas BBB P-glycoprotein/ABCB1's role in the brain clearance of Aβ has been demonstrated in both *in vitro* and transgenic AD models [16-20]. It is important to note that shuttling of Aβ across the BBB occurs by receptor/transporter-mediated processes that require the intact tertiary structure of the peptide that interacts with the carrier receptor(s).

Aβ brain intake and brain clearance have been studied using radioisotope-labeled Aβ peptides injected systemically or stereotactically into the brain, and by monitoring their appearance in various compartments, including cerebral spinal fluid (CSF) [21]. A molecular imaging tracer, [^11^C]-Pittsburgh compound B (PiB), which binds to Aβ plaques, has been used in small-animal and human PET (positron-emission tomography) imaging studies to monitor Aβ plaque load and its clearance in response to treatment [22]. The purpose of this study protocol is to demonstrate the utility of a simple and accessible *in vivo* optical imaging method for studying Aβ trafficking across the BBB in experimental animals in a dynamic, prospective fashion not achievable with radioactive tracers. Using this method, we demonstrated differences in Aβ trafficking across the BBB in animals deficient in two major ABC efflux pumps, mdr-1 P-glycoprotein/Abcb1 and Abcg2.

## Methods and design

### Materials

Synthetic human Aβ_1-40_ and scrambled Aβ_40-1_ peptides were purchased from Biopeptides Co., Inc (San Diego, CA, USA). Cy5.5 labeling kits (Cy5.5™ Mono NHS ester) and ECL Plus reagent kits were purchased from Amersham Biosciences/GE Healthcares (Buckinghamshire, UK). A mouse monoclonal anti-Aβ antibody 6E10 was purchased from the Covance Inc (Montreal, QC, Canada), and a goat anti-mouse secondary antibody conjugated with Alexa 568 and a HRP-conjugated donkey anti-mouse IgG antibody were purchased from the Santa Cruz Biotech Inc (Santa Cruz, CA, USA). Fluorescein-labeled lectin, *Ulex europeaus* agglutinin (UEA-I), was purchased from Vector Laboratories Inc (Burlington, ON, Canada). Fetal bovine serum (FBS) was purchased from Hyclone Inc (Logan, Utah, USA). Dulbecco’s phosphate-buffered saline (1X) (PBS) was purchased from GIBCO/Invitrogen (Invitrogen Inc., Grand Island, NY, USA). Autoradiography films were purchased from Mandel Scientific (Guelph, ON, Canada).

### Aβ peptides preparation and labeling

Aβ_1-40_ peptide used in this study for optical imaging/tracking is the most abundant Aβ peptide found in the cerebral vasculature and is more soluble than Aβ_1-42_ peptide. Aβ_1-40_ peptides (1 mg/vial) were dissolved in 250 μL of 10 mM NaOH, and then 12.5 μL of 1 M HEPES [4-(2-hydroxyethyl)-1-piperazineethanesulfonic acid] was added to bring the pH to 8.0. The peptides were divided into 2 tubes (0.5 mg/tube) and kept at −80°C. Because Aβ peptides are commonly present as beta sheet structure in solution, Western blot analyses of the mixtures were performed, and the majority of the peptides (>95%) were monomers with a small proportion of dimers (data not shown). Aβ_1-40_ or scrambled Aβ_40-1_ peptides (0.5 mg, molecular weight 4329.86D) were labeled with the near-infrared fluorescent dye Cy5.5 (molecular weight 1128.42D) using the labeling kit (Cy5.5™ Mono NHS ester) as per manufacturer’s instructions [8].

Cy5.5 is a monofunctional dye with absorbance at 675 nm, extinction maximum of 250,000 M^-1^ cm^-1^, and emission maximum of 694 nm. The functional group commonly used for labelling peptides and proteins is the primary amino group provided by lysine or the N-terminal amino group. The labelling with Cy5.5 NHS ester utilizes acylation reaction at the amino group. The N-terminal amino group and two lysine residues present in both Aβ_1-40_ and scrambled Aβ_40-1_ peptides may be accessible to labelling with Cy5.5 dye. Thus, Aβ_1-40_ peptides can be efficiently labelled with Cy5.5 and then purified free from unincorporated dye for optical imaging. The Cy5.5-labeled peptide can be either injected into the systemic circulation or into the brain to monitor its transport across the BBB.

Aβ peptides (0.5 mg peptide) were added to 40 μL of carbonate buffer (pH 9.1) and 20 μL of Cy5.5 NHS Ester dye (200 μg in DMSO) and incubated in the dark with rotation at room temperature for at least 2 h. The molecular weight of a labeled Aβ peptide is up to 7715 Dalton. The labeled peptides were purified using a column Microcon Ultracel YM-3 (Regenerated cellulose 3000 MWCO, Millipore Corp., Bedford, MA, USA) to remove unincorporated Cy5.5. The amount of labeled peptides was quantified using a BCA Protein Assay kit (Thermo Scientific, Rockford, IL, USA) following the manufacturer’s instructions and the labeling efficiency was determined by the BioTek FL × 800 microplate reader (673 nm for excitation and 692 nm for emission). The labeling efficiency/molar ratio was two-three Cy5.5 molecules per Aβ peptide, and was the same for Aβ_1-40_ and the scrambled Aβ_40-1_. The purified Aβ peptides (100 μg in 100 μL) were diluted with 100 μL saline to a final volume of 200 μL and injected intravenously into mice.

### Aβ-Cy5.5 conjugate stability in serum

To evaluate Aβ-Cy5.5 conjugate stability in serum, the labeled peptide (5-μL volume containing ~1 μg Aβ) was added to either 35 μL of (non-inactivated) FBS or 35 μL of 1 × PBS (1: 8 dilution) and incubated at 37°C for 0, 0.5, 1, 2, 4, 6, and 8 hours, respectively. The peptides (4 μL) from each of the above reactions (40 μL/reaction) were added to the loading buffer, boiled for 10 min, and resolved on a 16% Tricine-SDS-PAGE as described [23]. The tricine-SDS-PAGE gel was scanned in the optical imager; the peptides in the gel were then transferred to a PVDF membrane for immuno-blotting [8]. A mouse monoclonal anti-human Aβ antibody 6E10 (1:1000 dilution) and the secondary HRP-conjugated donkey anti-mouse IgG antibody (1:5000 dilution) were used for immunodetection. ECL plus detection reagents were applied to the blots and the blots were exposed to autoradiography films.

### Animals

The experiments with animals have been approved by the Animal Care Committee of the National Research Council of Canada - Ottawa (NRC). Wild-type (wt), mdr-1a/b (Abcb1a/b) knockout (Abcb1^KO^), and Abcg2^-KO^ mice of FVB background were purchased from the Taconic Farms Inc (New York, USA) and maintained in the NRC Animal Facility at Ottawa. Pairs of adult wild-type mice and Abcb1^-KO^ and pairs of adult wild-type and Abcg2^-KO^ mice of the same body weight and same sex were matched for injections and imaging experiments. After initial testing of fluorescence signal with various injected doses of Cy5.5-labeld Aβ peptides, the optimal dose selected for the experiments was 100 μg of labelled peptide in 200-μL volume. The mice were injected via tail vein with free Cy5.5 dye (~78 μg in 200 μL volume) or Cy5.5-labeled Aβ_1-40_ (100 μg in 200 μL volume) or Aβ_40-1_ peptides (100 μg in 200 μL volume) and were imaged in eXplore Optix 670 (GE Healthcare Systems/ART Inc) at different time-points after the injection as described below.

### Time-domain *in vivo* optical imaging

One week before the experiments, animals were placed in cages with bedding that, if ingested, does not produce *in vivo* autofluorescence. The animals were anesthetized with inhaled isoflurane (4% for induction and 1.5% for maintenance) and the fur was shaved from the head and dorsal side of the body. The labeled peptides (100 μg) or Cy5.5 free dye (~78 μg) were injected intravenously (i.v.) via the tail vein. The animals were imaged at 2, 4, 6, and 8 h post-injection using the time-domain optical imager eXplore Optix 670 (GE Healthcare Systems/ART Inc). The imaging protocols were described in detail previously [8,24-27].

Briefly, each animal was positioned on a platform (dorsal side facing up) that was then placed on a heated plate (at 36°C) in the imaging system. The whole-body scan or selected region of interest (ROI) scan (i.e., head) was performed as described [25,27]. In all imaging experiments, a 670-nm pulsed laser diode with a repetition frequency of 80 MHz and a time resolution of 12 ps was used for excitation. The fluorescence emission at 700 nm was collected by a highly sensitive photomultiplier tube offset by 3 mm for diffuse optical topography reconstruction. The optical imager uses a Time-Correlated Single Photon Counting (TCSPC) detection system coupled with a pulsed laser source. Images are built point per point in a raster scan fashion. The combination of a raster-scanning approach with a pulsed laser excitation reduces background and allows for depth probing. A pulsed light source and time-resolved detection allows the system to resolve the nanosecond timescale of fluorescence emission. Each scanned point acquired with the system contains a photon time-of-flight distribution (also called a Temporal Point Spread Function or TPSF). Laser power and counting time per pixel were optimized at 60 mW and 0.5 seconds, respectively. The values remained constant during the entire experiment. The raster scan interval was 1.5 mm and was held constant during the acquisition of each frame, and 1,024 points were scanned for each ROI. The data were thus recorded as TPSF and the images were reconstructed as fluorescence concentration maps. Average fluorescence concentration data from ROI placed around the heads were subsequently analyzed using the software ART Optix Optiview (ART Inc., Montreal, QC, Canada). The software normalizes all images obtained in the same experimental run (i.e., paired animals, same injected solution) to the same fluorescent scale (expressed in arbitrary units). After the last scan, the mice were cardiac-punctured and then perfused transcardially with 50-mL cold saline with a peristaltic ISMATECH pump (IDEX Health & Science GmbH. Germany) at 5 mL/min for 10 min to wash out the remaining blood and circulating fluorescence. Brains were then extracted and scanned *ex*-*vivo* for fluorescence concentration

### Immunohistochemistry

To demonstrate the presence of Aβ peptides in the brain, the brains extracted at the end of the imaging protocol were frozen-sectioned at 10 μm and immunostained with a mouse monoclonal anti-human Aβ antibody 6E10 and a goat anti-mouse secondary antibody conjugated with Alexa 568 as described [3,4,8]. The sections were also counter-stained with fluorescein-labeled lectin, *Ulex europeaus* agglutinin (UEA-I), as described [28] to visualize cerebral vessels.

### Statistical analysis

The fluorescent concentrations in mouse brains were compared by one-way ANOVA followed by Newman-Keuls post-hoc test.

## Results

### Is Cy5.5 a substrate for mdr-1 P-glycoprotein or ABCG2?

**Figure 1:**
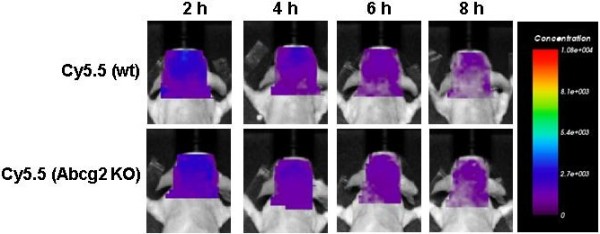
**Serial images of the concentration of the near-infrared fluorescent tracer Cy5.5 in the head region (ROI) after intravenous injection in wild-type and Abcg2**^**-KO **^**mice.** Cy5.5 free dye (~78 μg in 200 μL saline) was injected i.v. and mice were repeatedly imaged at 2, 4, 6 and 8 h using the eXplore Optix 670. The images shown were analyzed using ART Optix Optiview software and are representative of 4 animals per group.

To enable prospective *in vivo* optical imaging of the distribution of peripherally-injected Aβ peptides, the peptides were labeled with the near-infrared fluorescent dye Cy5.5. Since the principal aim of the present study was to monitor brain distribution of Cy5.5-labeled Aβ peptide in mice lacking major ABC transporters, the fluorescent tracer itself should not be the substrate for these transporters. To compare the permeability of BBB for Cy5.5 in wild-type, Abcb1^-KO^ and Abcg2^-KO^ animals, equal amounts of Cy5.5 tracer were intravenously injected into two pairs of wild-type and knockout mice; concentration of Cy5.5 fluorescence in their heads was determined by prospective optical imaging between 2 and 8 h after injection. The plasma half-life of Cy5.5 is about 30 min and the majority of the dye is cleared from the body in 2 hours. Remaining fluorescence in the head ROI was close to background and was not different between wild-type and Abcg2^-KO^ (Figure 1) or Abcb1^-KO^ (not shown) animals. Data indicate that the BBB in both wt and ABC-knockout animals is equally restrictive to Cy5.5, consistent with its molecular weight (1128.42D) and our previous observation that Cy5.5 can be detected in the brain only after the BBB breakdown [24,25]. Furthermore, since the deficiency in either mdr-1 P-glycoprotein (Abcb1) or Abcg2 has been shown not to affect BBB tight-junctions/passive permeability [29], the absence of brain accumulation of systemic Cy5.5 in these animals indicated that Cy5.5 is not a substrate for these transporters and can be used as fluorescent imaging tracer for Aβ tracking after systemic injection.

### Stability of Aβ-Cy5.5 conjugates in serum

**Figure 2:**
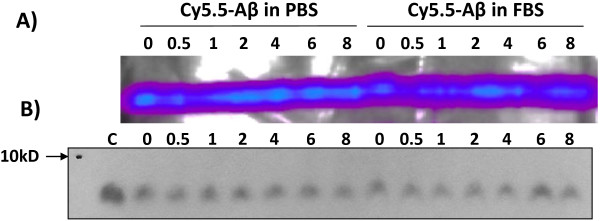
**The stability of Cy5.5-labeled Aβ**_**1-40 **_**peptide in serum *ex vivo*.** Cy5.5-labeled Aβ_1-40_ peptides were incubated in either phosphate buffered saline (PBS) or fetal bovine serum (FBS) at 37°C for indicated periods of time, resolved on a 16% Tricine-SDS-PAGE gel, imaged using eXplore Optix optical imager (**A**), and then blotted to PVDF membrane and probed with the anti-human Aβ antibody 6E10 (**B**). The lane C contains 1 μg unlabeled Aβ peptide; all other lanes contain ~1 μg Cy5.5-labeled Aβ peptides.

The stability of Aβ-Cy5.5 conjugates in serum was evaluated *ex vivo* by exposing conjugates to the intact, non-inactivated FBS or PBS for up to 8 h at 37°C. The dilutions (1:8 v/v) of the Aβ-Cy5.5 conjugates in FBS and PBS were adjusted to represent circulatory dilution after i.v. injection of 200 μL Aβ-Cy5.5 conjugates into adult mouse. Cy5.5-labeled Aβ peptides resolved on a tricine-SDS-PAGE gel were imaged in eXplore Optix, showing the presence of Cy5.5-signal after the exposure to either FBS or PBS for up to 8 h (Figure 2A). Immunoblots of the same tricine-SDS-PAGE gels using 6E10 anti-Aβ antibody (Figure 2B), showed single bands with similar mobility as unlabeled Aβ. Although the resolution of gels was not sufficient to resolve differences in MW (1–3 kD) between Cy5.5-labeled and unlabeled Aβ, no appreciable reductions of intact Aβ peptide bands were observed after incubation in either PBS or FBS, suggesting that Aβ-Cy5.5 conjugates were mostly intact in the serum *ex vivo* up to 8 hours.

### Brain accumulation of Aβ_1-40_ and scrambled Aβ_40-1_

**Figure 3:**
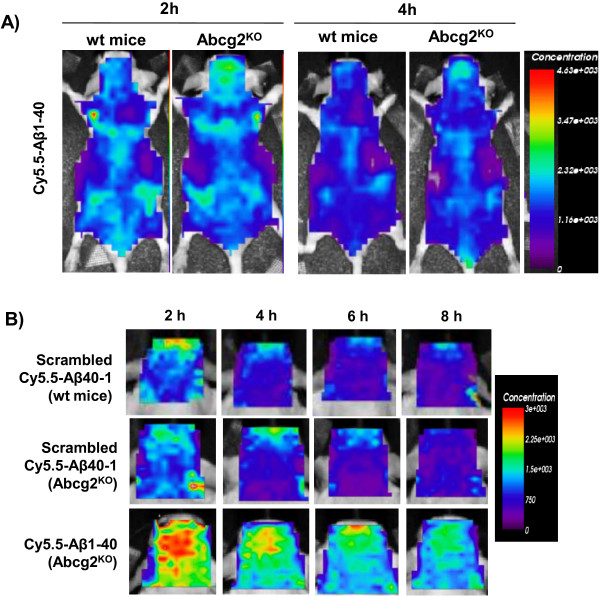
**Serial concentration images of Abcg2**^**-KO **^**and wild-type mice injected i.v. with either Cy5.5-labeled scrambled Aβ**_**40-1 **_**or Aβ**_**1-40**_**peptides.** The peptides (100 μg in 200 μL volume) were injected i.v. and whole body and head ROIs of animals were imaged at 2, 4, 6, and 8 h using the eXplore Optix 670. Panel **A** shows the whole body (dorsal) images of wild-type and Abcg2^-KO^ mice 2 and 4 h after i.v. injection of Cy5.5- Aβ_1-40_. Panel **B** shows head ROI fluorescence concentration images over time in wild-type mice injected with scrambled Aβ_40-1_, and Abcg2^-KO^ mice injected with either Cy5.5-labeled scrambled Aβ_40-1_ or with Cy5.5-labeled Aβ_1-40_ peptide. The images shown were analyzed with ART Optix Optiview software and are representative of 4 animals per group.

The biodistribution and systemic elimination (pharmacokinetics) of Aβ-Cy5.5 was evaluated by serial whole-body imaging after i.v. injection of labeled peptides into wild-type and transporter knockout animals. Our recent work demonstrated that the fluorescence residence time evaluated by whole-body imaging correlates closely with the circulation half-life of injected Cy5.5-labeled proteins [30]. The elimination kinetics of injected Aβ-Cy5.5 were similar in the wild-type and Abcg2^-KO^ (Figure 3A) and Abcb1^-KO^ (not shown), showing almost complete disappearance of fluorescence from the body between 2 h and 4 h after injection. The only discernible difference was the increased head fluorescence signal in transporter KO animals (Figure 3A).

Another important control for this study was to determine whether the observed accumulation of Cy5.5-labeled Aβ_1-40_ in the head region of KO animals was Aβ_1-40_. Therefore, Cy5.5-labeled scrambled Aβ_40-1_ was used in comparative experiments. After systemic injections of the equimolar concentrations (and equal fluorescence intensity) of Cy5.5-labeled peptides, the imaged head concentrations of scrambled Aβ_40-1_ were similar in wild-type and Abcg2^-KO^ (Figure 3B) or Abcb1^-KO^ mice (data not shown), while concentrations of Aβ_1-40_ were consistently higher than those of scrambled Aβ_40-1_ in Abcg2^-KO^ mice (Figure 3B). These observations suggested that only Aβ_1-40_, but not its scrambled version, is trafficked from the circulation into the brain, likely through binding to specific brain endothelial receptors/transporters.

### Brain accumulation of blood-borne Aβ_1-40_ peptides in Abcg2- or Abcb1- knockout animals

To evaluate whether there are differences in brain accumulation of blood-borne Aβ_1-40_ between wild-type and ABC-transporter-deficient animals, four pairs of adult wild-type and Abcb1^-KO^ mice and five pairs of adult wild-type and Abcg2^-KO^ mice were intravenously injected via the tail vein with the same amount of Cy5.5-labeled Aβ_1-40_ peptides and imaged prospectively over 2–8 h period. At the end of the protocol, mice were perfused with 50-mL cold saline and their brains were also imaged *ex vivo*.

The circulation half-life of injected ^125^I-Aβ peptides is about 35–45 min [31,32]. Therefore, the initial imaging time point of 2 hours (3–4 half-lives) was chosen to allow for a substantial clearance of the tracer from the circulation (also shown in Figure 3A). Therefore, fluorescence concentrations measured in the head ROI are assumed to represent mostly non-circulatory tracer, either bound/internalized into the brain vessels or transported into the brain parenchyma.

**Figure 4:**
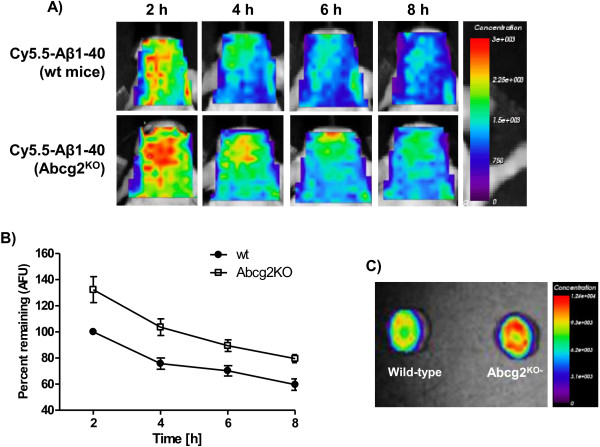
**Fluorescence concentration images of the head ROI after i.v. injection of 100-μg Cy5.5-labeled Aβ**_**1-40 **_**peptide into either wild-type or Abcg2**^**-KO **^**mice obtained by eXplore Optix 670.** Panel **A** shows representative serial concentration images of the head ROI at 2, 4, 6, and 8 h after Cy5.5-Aβ_1-40_ injection. The data are normalized to wild-type animals at 2 h as 100%. Panel **B** shows time-dependent fluorescence concentration changes (means ± SEM from four paired sets of experiments) in wild-type and Abcg2^-KO^ animals. The data was expressed as percent of fluorescence concentration normalized to 2-h wt animals and analyzed using one-way ANOVA and individual groups were compared Newman-Keuls post-hoc test (for wild-type vs. Abcg2^-KO^ mice: 2 h vs. 2 h p < 0.01, 4 h vs. 4 h p < 0.05, 6 h vs. 6 h p > 0.05, and 8 h vs. 8 h p > 0.05; for Abcg2^-KO^ vs. Abcg2^-KO^ mice: 2 h vs. 4 h p < 0.01, 2 h vs. 6 h p < 0.001, and 2 h vs. 8 h p < 0.001; for wild-type vs. wild-type mice: 2 h vs. 4 h p < 0.05, 2 h vs. 6 h p < 0.05, and 2 h vs. 8 h p < 0.01). Panel **C** shows *ex*-*vivo* brain images from animals sacrificed 8 h after the Cy5.5-Aβ_1-40_ injection by transcardial perfusion.

Comparisons of fluorescent concentrations in the head ROIs indicated that the fluorescence concentration of the tracer is statistically higher (133%) in Abcg2^-KO^ mice compared to wild-type mice at each time point assessed (Figure 4A, B). However, fluorescence decay curves over 2–8 h (analyzed using one-phase exponential decay) indicated similar decay dynamics in Abcg2^-KO^ mice (t_1/2_ = 2.34 h) compared to wild-type (t_1/2_ = 1.97 h). Imaging of perfused brains *ex vivo* (Figure 4C), indicated that brain fluorescence levels remained elevated in Abcg2^-KO^ mice in comparison to wild-type animals 8 h after injection.

**Figure 5:**
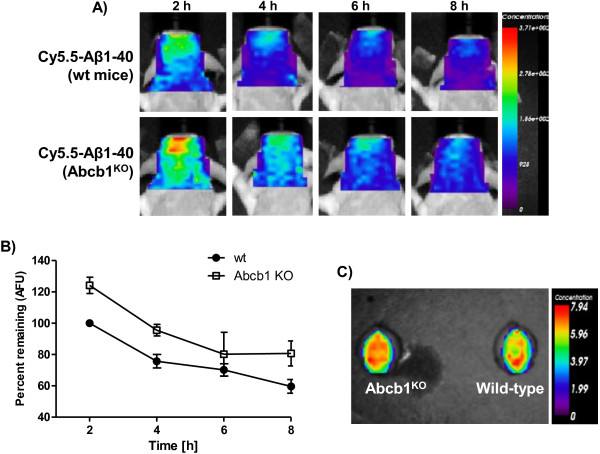
**Fluorescence concentration images of the head ROI after i.v. injection of 100-μg Cy5.5-labeled Aβ**_**1-40 **_**peptide into either wild-type or Abcb1**^**-KO **^**mice obtained by eXplore Optix 670.** Panel **A** shows representative serial concentration images of the head ROI at 2, 4, 6, and 8 h after Cy5.5-Aβ_1-40_ injection. The data are normalized to wild-type animals at 2 h as 100%. Panel **B** shows time-dependent fluorescence concentration changes (means ± SEM from four paired sets of experiments) in wild-type and Abcb1^-KO^ animals. The data was expressed as percent of fluorescence concentration normalized to 2-h wt animals and analyzed using one-way ANOVA and individual groups were compared Newman-Keuls post-hoc test (for wild-type vs. Abcb1^-KO^ mice: 2 h vs. 2 h p < 0.05, 4 h vs. 4 h p > 0.05, 6 h vs. 6 h p > 0.05, and 8 h vs. 8 h p > 0.05; for Abcb1^-KO^ vs. Abcb1^-KO^ mice: 2 h vs. 4 h p < 0.05, 2 h vs. 6 h p < 0.01, and 2 h vs. 8 h p < 0.001; for wild-type vs. wild-type mice: 2 h vs. 4 h p > 0.05, 2 h vs. 6 h p < 0.05, and 2 h vs. 8 h p < 0.001). Panel **C** shows *ex*-*vivo* brain images from animals sacrificed 8 h after the Cy5.5-Aβ_1-40_ injection by transcardial perfusion.

The head fluorescence concentrations in Abcb1^-KO^ mice was also significantly higher than in wild-type mice at the outset of imaging measurements (2 hours) (124.2% in Abcb1^-KO^ mice normalized to wild-type mice at 2 h as 100%) (Figure 5A, B). The fluorescence concentration ‘decay’ over 2–8 h, showed slightly faster decay dynamics in Abcb1^-KO^ mice (t_1/2_ = 1.38 h) compared to wt-type (t_1/2_ = 1.97 h) (Figure 5B). At the end of the imaging protocol perfused brains were imaged *ex*-*vivo* (Figure 5C), confirming that the fluorescence concentration differences observed *in vivo* were not due to circulating tracer.

### Immunohistochemistry detects Aβ peptides in mouse brain

**Figure 6:**
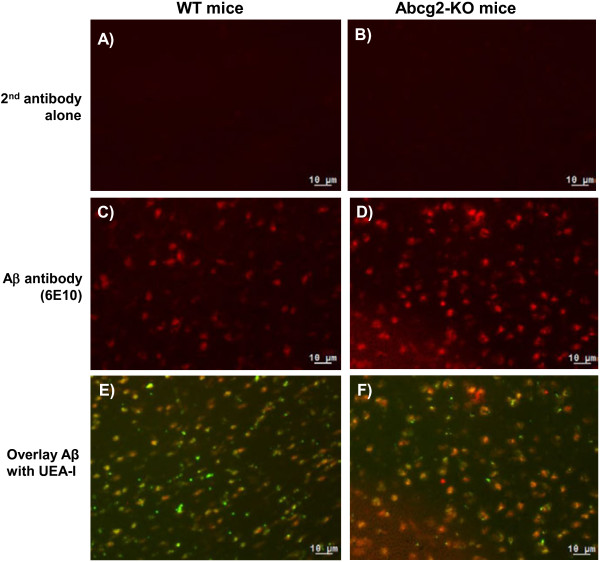
**Aβ**_**1-40 **_**immunohistochemistry in brain sections using a mouse monoclonal anti-Aβ antibody 6E10.** The wild-type and Abcg2^-KO^ mice were injected with 100 μg Cy5.5-labeled human Aβ_1-40_ peptides and brain tissues were collected 8 h post-injection. Brain sections were incubated with either the secondary antibody alone (panels **A** &**B**) or 6E10 followed with the Alexa 568 (red)-conjugated secondary antibody (panels **C** &**D**) and were co-stained with UEA-I (green) to visualize cerebral vessels (panels **E** &**F**). Images (20 × magnifications) are representative of results obtained from 3 animals in each group.

To determine whether measured Cy5.5 fluorescence in imaging experiments originated from the intact Cy5.5-Aβ_1-40_ conjugates rather than from the proteolytically-degraded fragments or dye alone, Aβ peptides were detected in the brain tissues of wild-type and Abcg2^-KO^ mice using an anti-Aβ antibody, 6E10. Brain sections probed with secondary antibody only (Figure 6A & B) showed no detectable signal. The immunoreactive Aβ (red) was detected in brain sections of both wild-type and Abcg2^-KO^ animals injected with Cy5.5-labeled Aβ_1-40_ peptides (Figure 6C & D). Aβ was observed co-localizing with brain vessels as well as within brain parenchyma (Figure 6E & F).

6E10 antibody recognizes human, but not murine (endogenous) form of Aβ peptides. In our previous study [33] investigating the expression of Aβ_1-40_ and Aβ_1-42_ in the brains of wild-type, Abcg2^-KO^, Tg-SwDI, and double transgenic Tg-SwDI/Abcg2^-KO^ mice up to 15 months of age, murine forms of Aβ peptides were below detection limits (Mouse Aβ ELISA kits, Invitrogen Inc), whereas human forms were detected in Tg-SwDI, and double transgenic Tg-SwDI/Abcg2^-KO^ mice. Therefore, the presence of immunoreactive Aβ in the mouse brain after i.v. injection of Cy5.5-labeled human Aβ peptides suggested that these peptides were blood-borne and confirmed that at least a portion of imaging signal originated from intact Aβ-Cy5.5 conjugates.

## Discussion

This study describes the application of prospective *in vivo* optical imaging protocols to study brain accumulation of systemically injected Aβ peptides in wild-type and animals deficient in specific transporters previously implicated in Aβ transport across the blood-brain barrier.

Radio-labeled [^125^I]-or [^3^H]-Aβ peptides have been used to study their BBB transport in animal models. The labelled peptides are either injected intravenously to analyze brain uptake or intra-cerebrally to investigate their clearance from the brain; animals are sacrificed at different time points and the radioactivity is determined in desired compartments. *In vivo* molecular imaging approaches that ‘track’ Aβ peptides non-invasively are dynamic methods that can be used for assessing Aβ levels in response to treatments. Notably, PET imaging with [C^11^]-PiB [*N*-methyl-[11C]2-(4-methylaminophenyl)-6-hydroxybenzothiazole] has been used for quantitative assessment of brain Aβ load in Alzheimer’s patients [34] and in APP/PS1 mouse [22]. Apart from requiring ‘on-site’ radioisotope labeling and access to expensive PET equipment, this approach is not applicable for ‘tracking’ peripheral Aβ peptides. Optical molecular imaging/tracking of Aβ peptides functionalized with the near-infrared imaging tracer is a viable alternative that can provide high sensitivity in experimental setting, although it does not have the quantification capabilities of PET. Among *in vivo* optical imaging systems, time-domain optical imaging has a clear advantage over Continuous Wavelength (CW) systems in that its pulsed laser source can penetrate skull to excite the fluorescent tracer in deep tissues. In contrast to CW systems where emitted light is collected by a CCD camera that cannot resolve the depth of the signal, with time-resolved imaging platform each collected photon retains time-of-flight distribution (also called a Temporal Point Spread Function or TPSF) from which depth (optical tomography), fluorescence concentration and fluorescence lifetime can be extracted [24-27]. This and other studies [35,36] have shown that this imaging method is a useful non-invasive approach to investigate Aβ transport, distribution, and clearance from the brain that complements other imaging approaches.

The aberrant transport and clearance of Aβ peptides across the BBB, mediated by a spectrum of receptors and transporters including RAGE, LRP-1, and members of ABC family, contributes to Aβ accumulation in the brain and in the cerebral vasculature [7,37,38]. ABC family members MDR-1 P-glycoprotein/ABCB1 and ABCG2/BCRP are two major drug efflux transporters located at the luminal surface of the BBB [39,40]. In mice, mdr-1a (Abcb1a) is the primary drug efflux transporter expressed at the BBB; while mdr-1b (Abcb1b) is the main isoform detected in the brain parenchyma [41]. Murine mdr-1 P-glycoprotein is encoded by both *mdr*-*1a* (*Abcb1a*) and *mdr*-*1b* (*Abcb1b*), which share 90% sequence homology and have 80% homology to human *MDR1* (*ABCB1*). The mdr-1a/b (Abcb1a/b) double knockout completely eliminates mdr-1-mediated transport activity at the BBB. Several published studies [8,15-20] presented the evidence that inhibition or deficiency of Abcg2 or mdr-1 P-glycoprotein increases Aβ intake in cell models and reduces brain Aβ clearance in animal models.

To further evaluate the roles of Abcb1 and Abcg2 in Aβ trafficking across the BBB, we developed the non-invasive optical imaging method for ‘tracking’ systemically injected fluorescently-labeled Aβ peptides in Abcb1^-KO^ and Abcg2^-KO^ mice. For the purpose of *in vivo* tracking Aβ peptides were conjugated to the near-infrared optical fluorescence tracer Cy5.5. Since Aβ degrading proteases including insulin degrading enzyme (IDE), angiotensin converting enzyme (ACE) and neprilysin [42,43] are active in the blood and can contribute to Aβ degradation, the stability of Cy5.5-Aβ conjugates in serum over 8 hours was confirmed *ex*-*vivo*, proving that the optical signal in imaging experiments originated predominantly from intact Cy5.5-Aβ conjugates. Imaging assessment of the whole-body biodistribution and elimination kinetics of Cy5.5-Aβ peptides, demonstrated similar elimination kinetics in wild-type and KO animals; the majority of peripheral tracer was eliminated by 2–4 h after the injection. This is in agreement with previous studies that reported the circulation half-life of injected [^125^I]-Aβ peptides of about 35–45 min; ~81% of the injected Aβ was cleared from blood by 60 min after administration in adult monkey [32,33,44].

Head ROI imaging protocols were initiated 2 hours after tracer injection, allowing 3–4 circulation half-lives; therefore, measured head fluorescence concentration was primarily indicative of the brain-accumulated/retained tracer, with small contribution of circulating tracer. In both Abcb1^-KO^ and Abcg2^-KO^ animals, brain tracer concentration was higher than in the wild-type animals at 2 hours, suggesting that any of the following processes or their combination might have been altered in knockout animals: a) the rate of Aβ brain influx was increased; b) the rate of Aβ brain elimination was slower; and c) Aβ binding/uptake into brain vessels was increased. Based on the current data, we cannot exclude any of these processes being responsible for the observed tracer concentration differences at 2 hours after injection. However, given the relatively short circulation half-life of Aβ, we can assume that imaging measurements between 2 and 8 hours after injection reflect predominantly brain elimination kinetics of Aβ. Brain-injected [^125^I]-Aβ_1-40_ peptide has been shown to clear rapidly via receptor-mediated transport with *t*_1/2_ of 25 minutes [45]. A single photon emission computed tomography (SPECT) study in squirrel monkeys [46], demonstrated a bi-phasic brain clearance of intracerebrally microinfused [^123^I]-Aβ_1-40_, with short t_1/2_ ranging from 1.1 ~ 2.7 hours and accompanying plasma appearance of [^123^I]-Aβ_1-40_, suggesting active brain-to-blood transport. Comparisons of Aβ fluorescence decay curves between 2 and 8 h in wild-type and ABC-transporter knock-out animals indicated similar fluorescence decay (elimination) kinetics within the range of clearance rates described by Bading *et al*[46]. Due to limited number of imaging time-points and the study design, it was not possible to discern whether the observed elimination kinetics of Aβ are due to active reverse transport across the BBB or to the interstitial fluid bulk-flow clearance.

Whereas lack of Abcg2 in this study did not appear to affect the rate of Aβ elimination from the brain, it resulted in higher initial accumulation of injected Aβ, suggesting that it has a role in either limiting brain access of circulating Aβ or mediating fast brain elimination phase of Aβ, or both. In agreement with our observations, a recent study [15] using the *in situ* brain perfusion technique showed that GF120918, a dual inhibitor of Abcb1 and Abcg2, strongly enhanced the uptake of [^3^H]-Aβ_1-40_ in the brains of Abcb1-deficient mice, but not in the brains of Abcb1/Abcg2-deficient mice. ABCG2 is up-regulated in human AD brain with cerebral amyloid angiopathy (CAA) [8] where it modulates Aβ-induced vascular oxidative stress [33,47].

Similarly, the deficiency of mdr-1/P-glcoprotein significantly increased brain accumulation of systemically injected Aβ but also slightly accelerated its elimination from the brain. This observation is consistent with some previously reported studies. Deposition of Aβ peptides has been found to inversely correlate with MDR-1 P-glycoprotein/ABCB1 expression in the brains of elderly non-demented humans as well as in the brains of Alzheimer’s patients [37,48,49]. In addition, Aβ was found to down-regulate BBB mdr-1 P-glycoprotein (Abcb1) expression in mice [50]. Cirrito and colleagues [17] demonstrated that Aβ removal from the brain was partially mdr-1-dependent in mdr-1a/b KO mice. Furthermore, restoration of mdr-1 P-glycoprotein/Abcb1 at the BBB by PXR (Pregnane X Receptor) agonist reduced brain Aβ load in a mouse model of Alzheimer's disease [18].

The definitive interpretation of data provided in this study is confounded by possible activation of compensatory mechanisms in knock-out animals. For example, the Abcb1/P-glycoprotein-null mice were found to have lower brain expression of LRP-1 compared to wild-type mice [17]. We found no compensatory changes in Abcb1a/mdr-1a and Abcb1b/mdr-1b expression in the brains of Abcg2^-KO^ mice (data not shown); however, we cannot ascertain whether other Aβ transporters (i.e., RAGE, LRPs) were specifically affected in brain endothelial cells in Abcb1- or Abcg2^-KO^ animals.

Pharmacological studies using selective inhibitors of BBB transporters in cell systems [15,20] provided strong evidence that both ABCB1/MDR-1 P-glycoprotein and ABCG2 have the capacity to interact with and shuttle Aβ across cellular membranes. *In vivo* imaging studies, including ours presented here, support this notion and provide means for dynamic analyses of integrative influences of BBB transporters on Aβ trafficking in and out of the brain.

In summary, this study protocol describes potential application of time-domain prospective *in vivo* imaging in assessing BBB trafficking of systemically injected compounds, including Aβ peptides, labeled with near-infrared fluorescent imaging tracers. The protocol is particularly useful in assessing BBB trafficking of such compounds in animals exhibiting modifications of various BBB transporters, such as for example gene knock-out or over-expression of ABC-family of efflux pumps. Similarly, this imaging method can be used to evaluate kinetics of brain elimination of intra-cerebrally-injected compounds as recently described in our study on FcRn-mediated brain elimination of fluorescently-labeled macromolecules [51].

## Abbreviations

AD: Alzheimer’s disease; BBB: Blood-brain barrier; BCRP: Breast Cancer Resistant Protein; CW: Continuous Wavelength; FBS: Fetal bovine serum; HEPES: 4-(2-hydroxyethyl)-1-piperazineethanesulfonic acid; KO: Knockout; LRP-1: Low-density lipoprotein receptor-related protein-1; MDR: Multi-drug resistance; PiB: Pittsburgh compound B; PET: Positron-emission tomography; RAGE: Receptor for Advanced Glycation Endproducts; ROI: Region of interest; TPSF: Temporal Point Spread Function; wt: Wild-type; UEA-I: Ulex Europeaus Agglutinin-I.

## Competing interests

The authors declare that they have no competing interests.

## Authors’ contributions

WZ conceived and designed the experiments, performed data analyses and prepared the figures, wrote and revised the manuscript. HX carried out most of the experiments and analyzed the data. AJ and KP assisted HX in performing the experiments. DC conducted brain tissue sections and IHC. HL and EB conducted in vitro Aβ stability assay. DF prepared and analyzed some of optical images. DS conceived the project and contributed to data analyses, figure preparation, writing and revising the manuscript. All authors have read and approved the final version of the manuscript.
